# Supplementary material for: Continuous tuning of persistent luminescence wavelength by intermediate-phase engineering in inorganic crystals
Source: Nat Commun. 2024 Aug 9;15:6797. doi: 10.1038/s41467-024-51180-5 (PMC11316030; doi:10.1038/s41467-024-51180-5)
Supplement: Supplementary file 1 — Supplementary Information [file 41467_2024_51180_MOESM1_ESM.pdf]

Supplementary Information

Continuous Tuning of Persistent Luminescence Wavelength by Intermediate-Phase Engineering in Inorganic Crystals

Xin Zhang[1,4], Hao Suo[1,2,4], Yang Guo[1], Jiangkun Chen[1], Yu Wang[2], Xiaohe Wei[1], Weilin Zheng[1], Shuohan Li[1], Feng Wang[1,3,*]

[1] Department of Materials Science and Engineering, City University of Hong Kong, Hong Kong SAR, China.

[2] College of Physics Science & Technology, Hebei University, Baoding 071002, China.

[3] Hong Kong Institute for Clean Energy, City University of Hong Kong, Hong Kong SAR, China.

[4] These authors contributed equally to this work.

*Correspondence: fwang24@cityu.edu.hk (F.W.).

I. Supplementary Figures

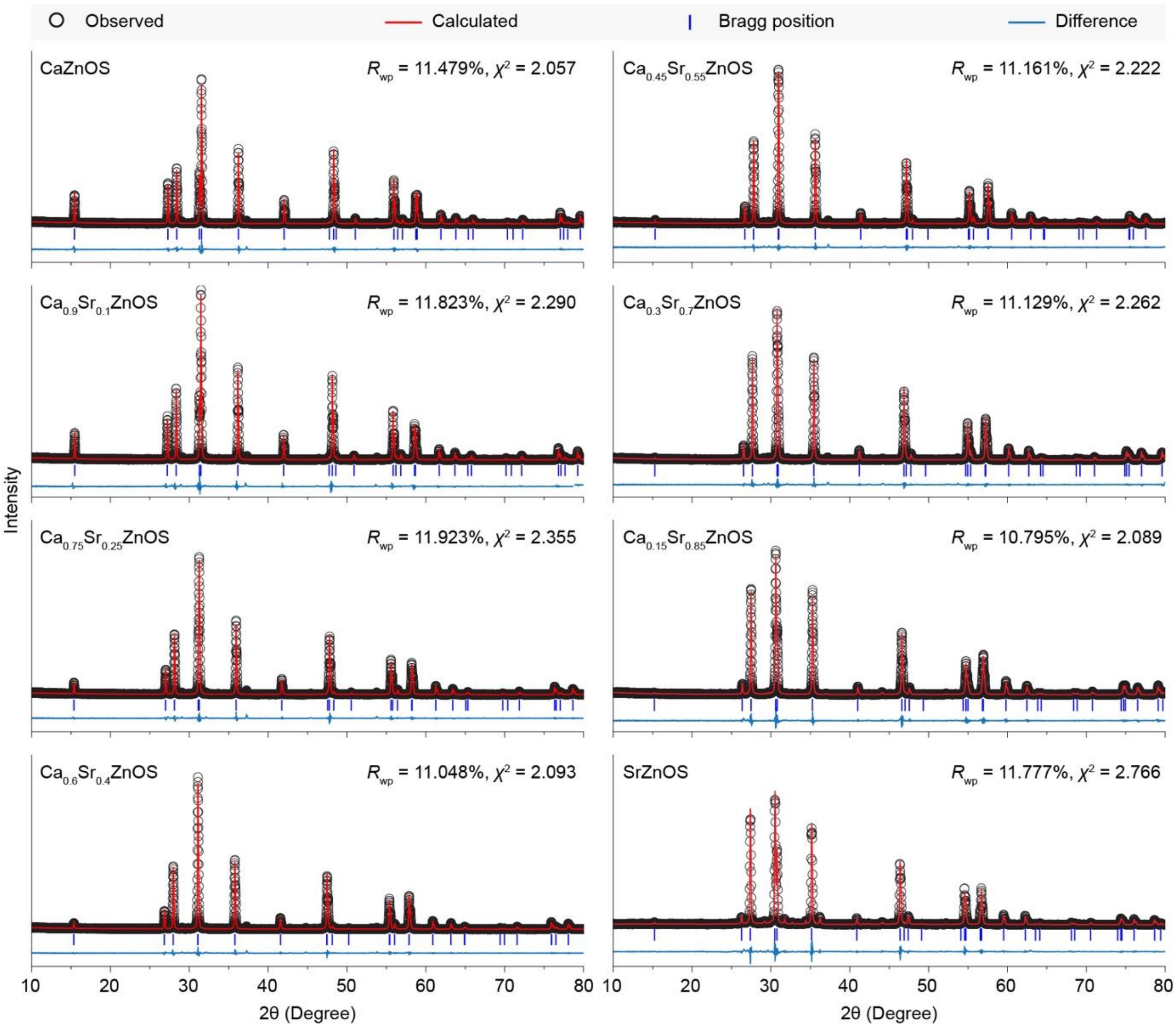

Supplementary Fig. 1. Rietveld refinement results of XRD patterns for $Ca_{1-x}Sr_xZnOS$:0.1%Cu^+/1%Y^{3+} crystals (x = 0, 0.1, 0.25, 0.4, 0.55, 0.7, 0.85, 1) sintered at 1348 K for 3 hours. The calculated crystallographic structural parameters of the corresponding samples are compiled in Supplementary Table 2.

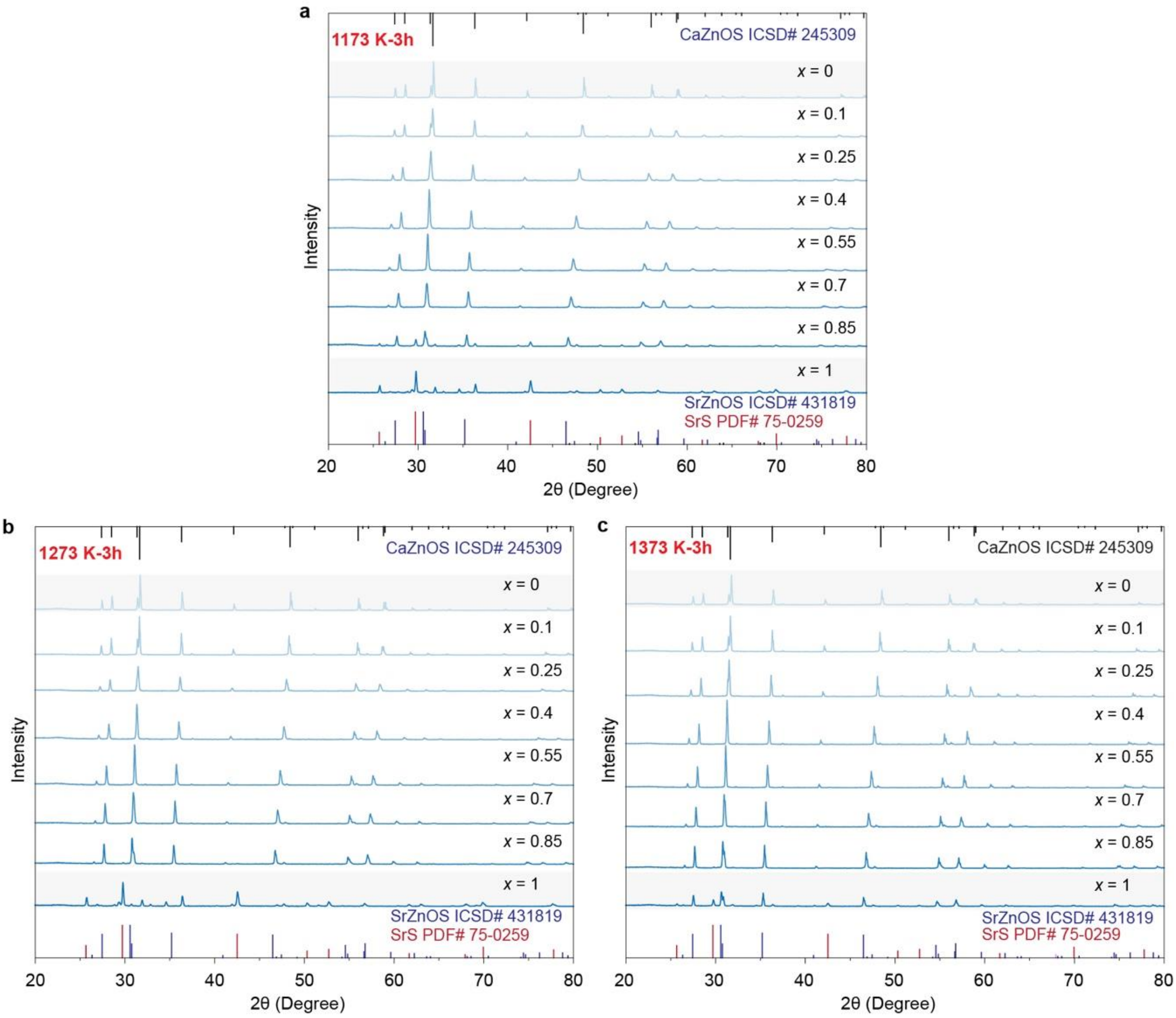

Supplementary Fig. 2. Powder XRD patterns of $Ca_{1-x}Sr_xZnOS$:0.1%Cu^+/1%Y^{3+} crystals (x = 0, 0.1, 0.25, 0.4, 0.55, 0.7, 0.85, 1) sintered for 3 hours at a) 1173 K, b) 1273 K and c) 1373 K, respectively. Except for SrZnOS, the series of $Ca_{1-x}Sr_xZnOS$:0.1%Cu^+/1%Y^{3+} crystals (x = 0, 0.1, 0.25, 0.4, 0.55, 0.7, 0.85) could be easily obtained in a wide temperature window (1173–1373 K) without further purification.

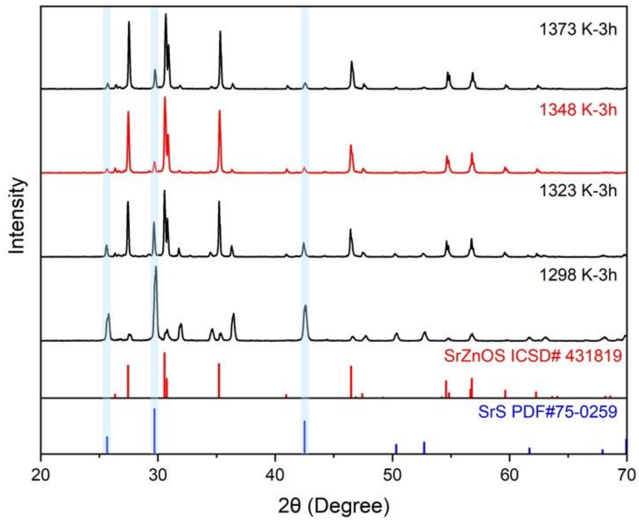

Supplementary Fig. 3. Powder XRD patterns of SrZnOS:0.1%Cu^{+}/1%Y^{3+} samples sintered at various temperatures. The SrZnOS phase forms only in a very narrow temperature range close to 1348 K.

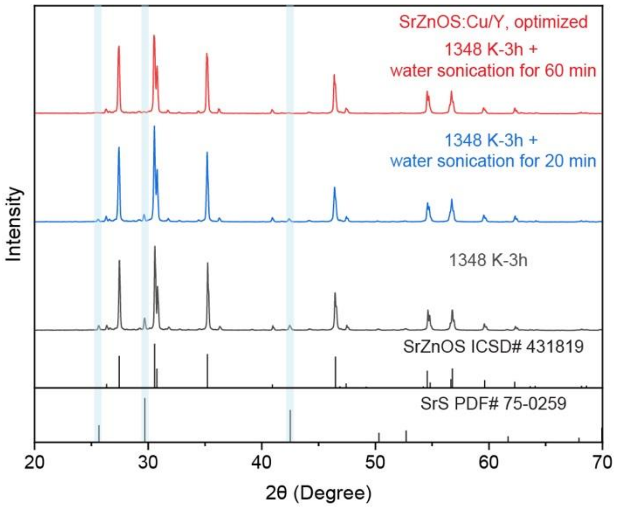

Supplementary Fig. 4. Powder XRD patterns of the SrZnOS:0.1%Cu^+/1%Y^{3+} sample (sintered at 1348 K for 3 h) before and after purification using water sonication. The synthesis procedure of pure-phase SrZnOS samples was tedious. The optimized SrZnOS:Cu/Y was obtained at the reaction temperature of 1348 K with an annealing time of 3 hours, followed by water sonication for 60 minutes.

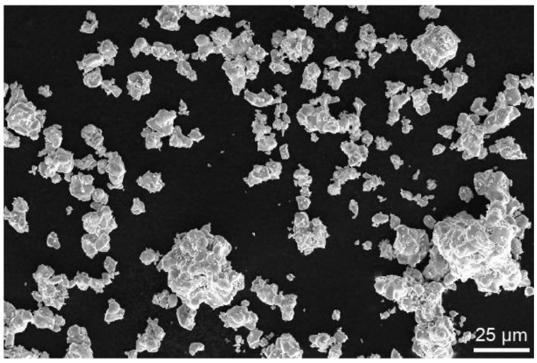

Supplementary Fig. 5. SEM image of the representative $Ca_{0.45}Sr_{0.55}ZnOS$:Cu/Y sample.

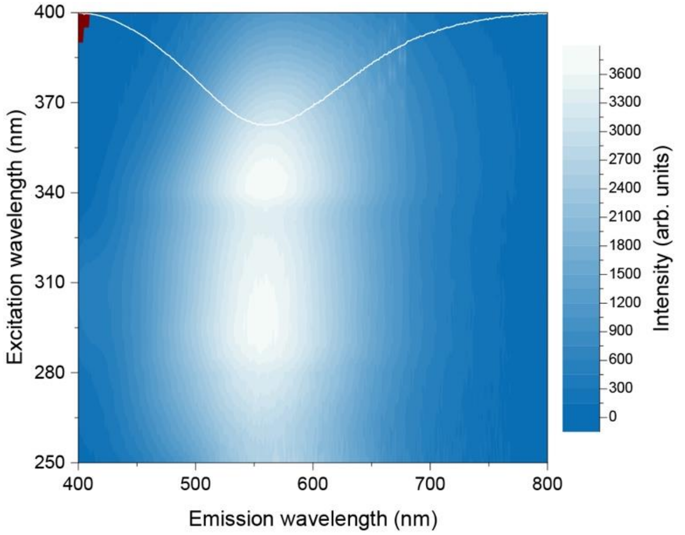

Supplementary Fig. 6. Contour plot of the excitation-dependent photoluminescence of the $Ca_{0.45}Sr_{0.55}ZnOS$:0.1%Cu^{+}/1%Y^{3+} crystal.

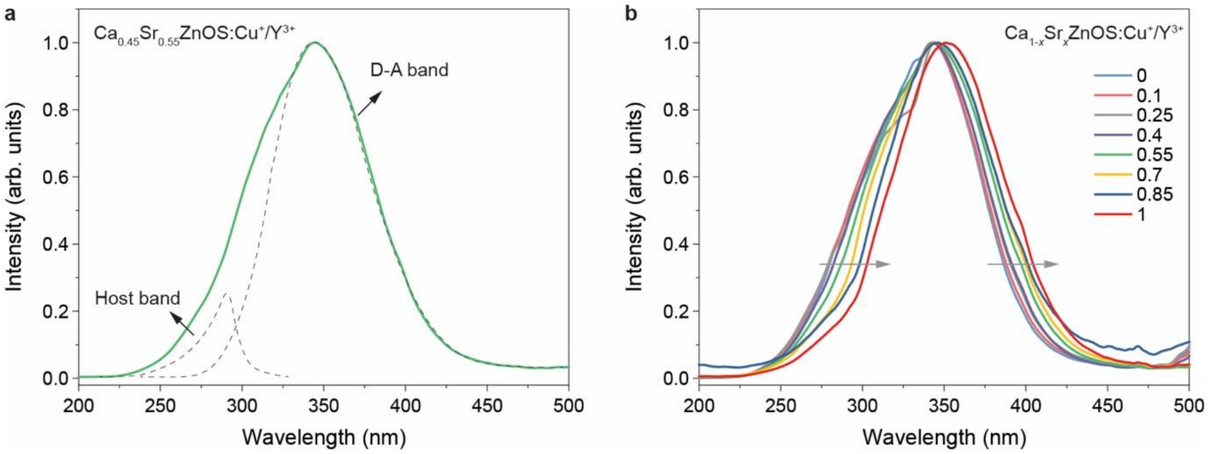

Supplementary Fig. 7. Photoluminescence excitation spectra of a) $Ca_{0.45}Sr_{0.55}ZnOS:0.1\%Cu^+/1\%Y^{3+}$ and b) the series of $Ca_{1-x}Sr_xZnOS:0.1\%Cu^+/1\%Y^{3+}$ crystals (x = 0, 0.1, 0.25, 0.4, 0.55, 0.7, 0.85, 1).

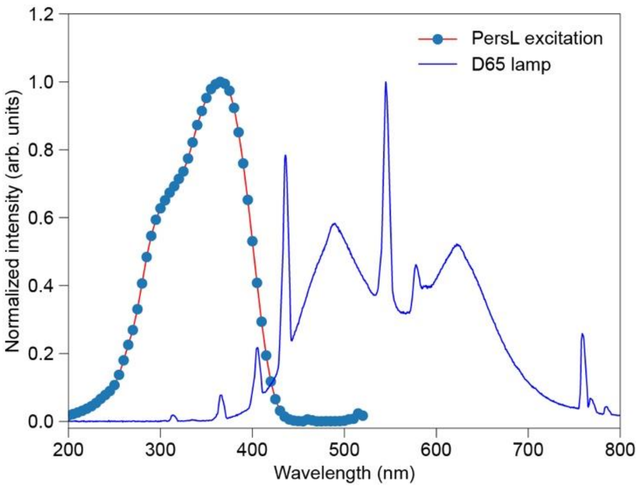

Supplementary Fig. 8. PersL excitation spectrum of CaZnOS:Cu/Y compared to the emission spectrum of a commercial D65 lamp.

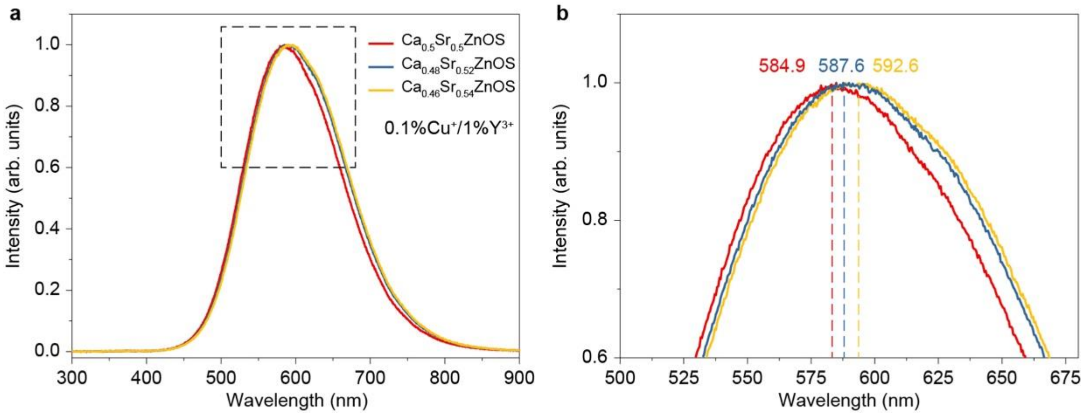

Supplementary Fig. 9. a) PersL spectra of the Ca(Sr)ZnOS:Cu^{+}/Y^{3+} crystals. b) Enlarged plot of the rectangular part in (a).

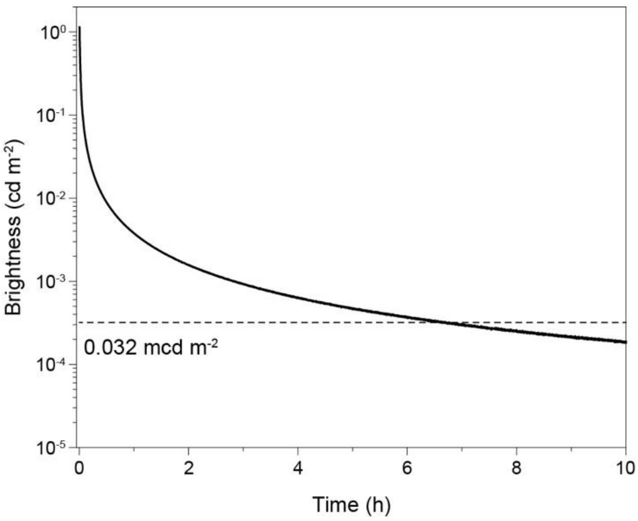

Supplementary Fig. 10. PersL decay curve of the $Ca_{0.6}Sr_{0.4}ZnOS$:0.1%Cu^{+}/1%Y^{3+} sample with the highest initial brightness. The PersL intensity was recorded by an FLS980 spectrometer and calibrated with a luminance meter (CHROMA METER CS-200).

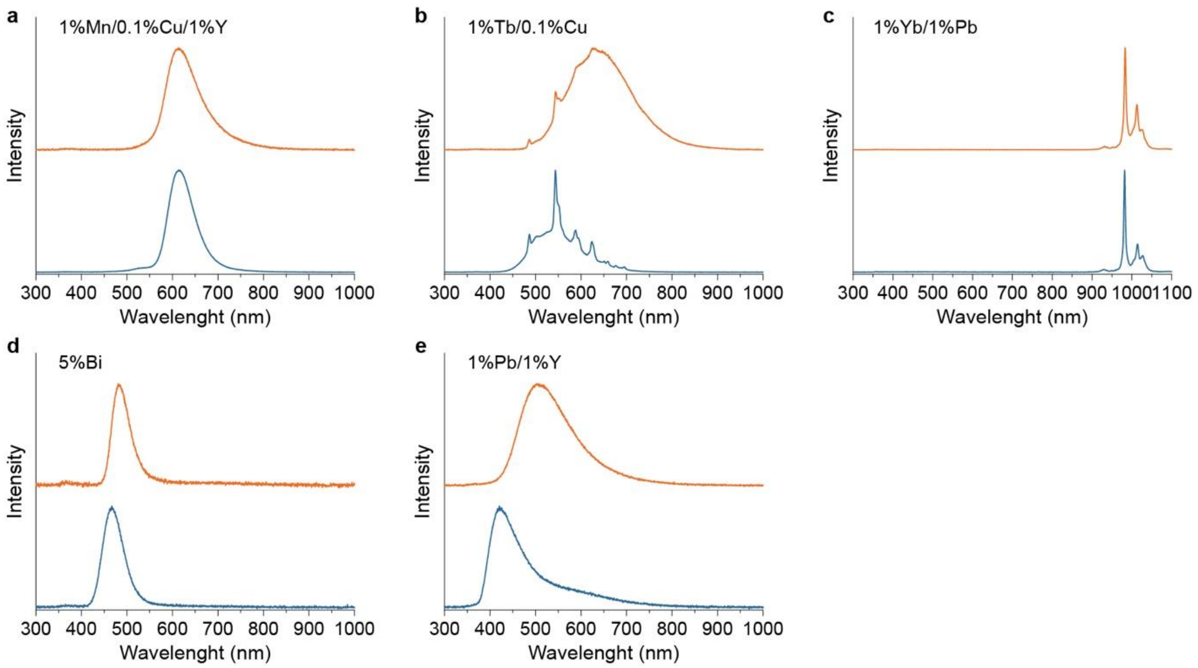

Supplementary Fig. 11. PersL spectra of CaZnOS (blue) and SrZnOS (orange) crystals doped with various activators: a) Mn^{2+}, b) Tb^{3+}, c) Yb^{3+}, d) Bi^{3+} and e) Pb^{2+} (charged @ 365 nm)[1].

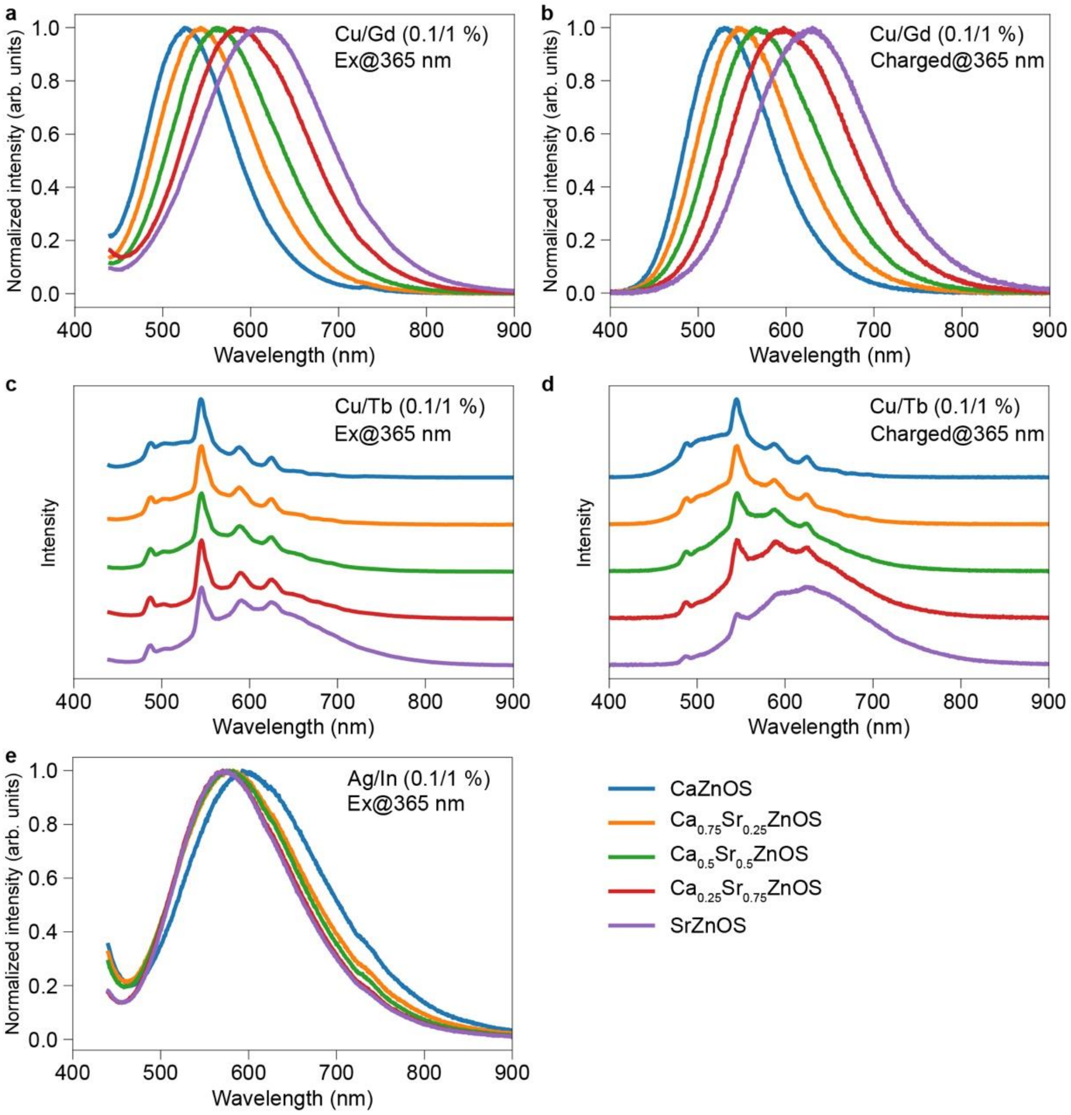

Supplementary Fig. 12. Tunable D-A luminescence in Ca(Sr)ZnOS crystals doped with various activators: a-b) Cu/Gd, c-d) Cu/Tb and e) Ag/In.

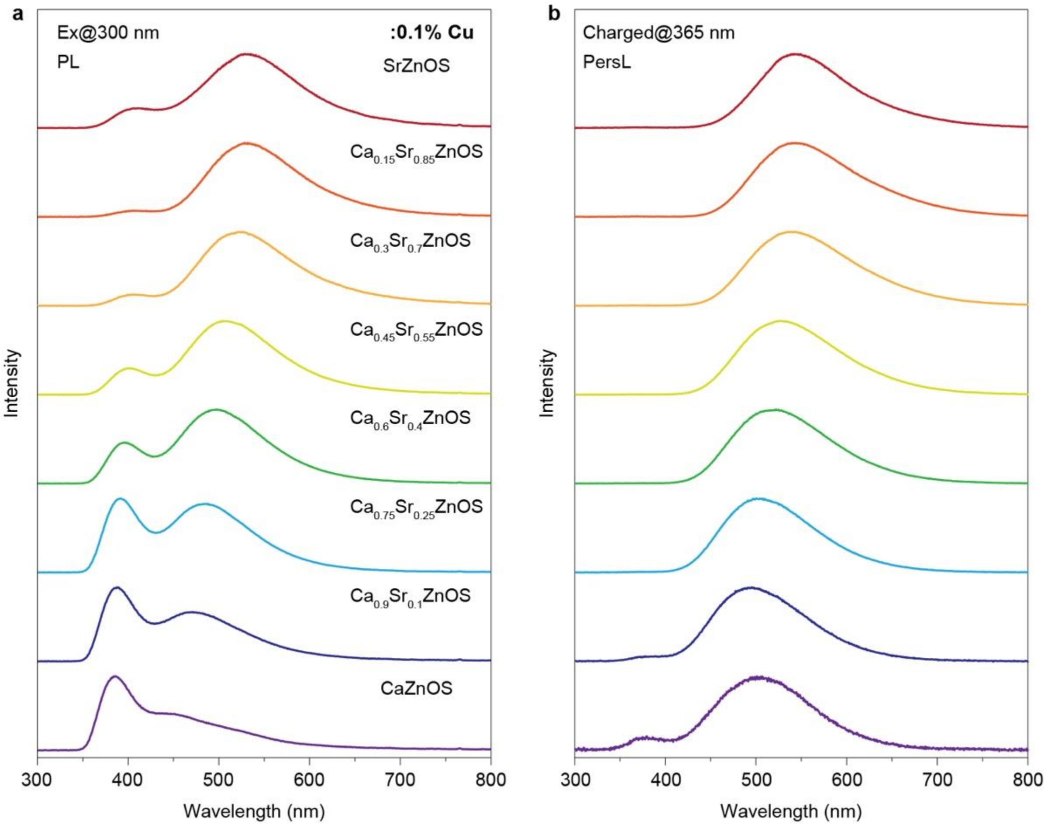

Supplementary Fig. 13. a) PL and b) PersL spectra of $Ca_{1-x}Sr_xZnOS$:0.1%Cu^+ (x = 0−1) crystals, revealing moderate tuning of PersL with D-A states induced by singly doping Cu^+.

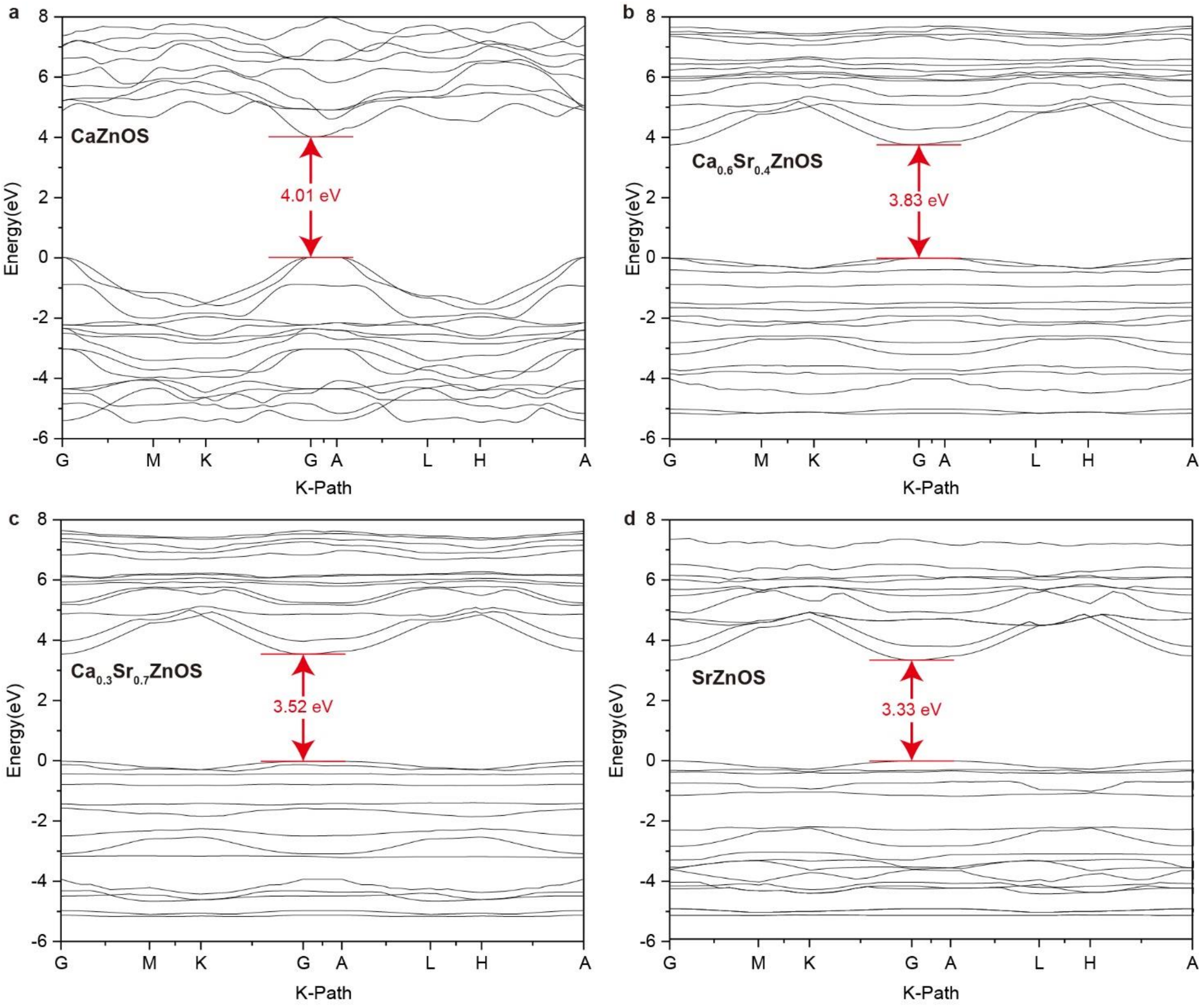

Supplementary Fig. 14. Calculated electronic band structures of a) CaZnOS, b) $Ca_{0.6}Sr_{0.4}ZnOS$, c) $Ca_{0.3}Sr_{0.7}ZnOS$ and d) SrZnOS.

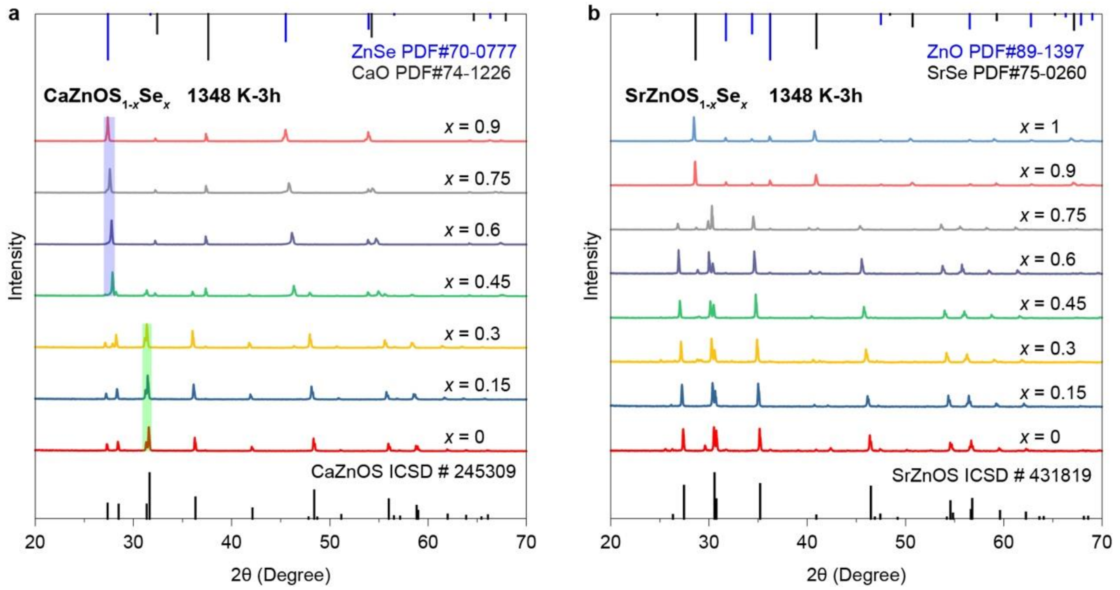

Supplementary Fig. 15. Powder XRD patterns of the nominal a) $CaZnOS_{1-x}Se_x$ and b) $SrZnOS_{1-x}Se_x$ samples. The attempt to synthesize single-phase CaZnOS(Se) or SrZnOS(Se) was unsuccessful.

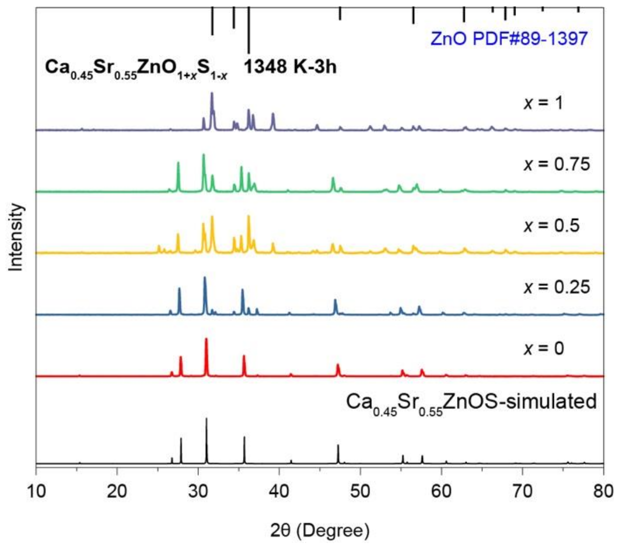

Supplementary Fig. 16. Powder XRD patterns of the nominal $Ca_{0.45}Sr_{0.55}ZnO_{1+x}S_{1-x}$ samples. The investigation into the effect of anions on D-A emission by varying the O content was hindered due to the inability to prepare single-phase samples.

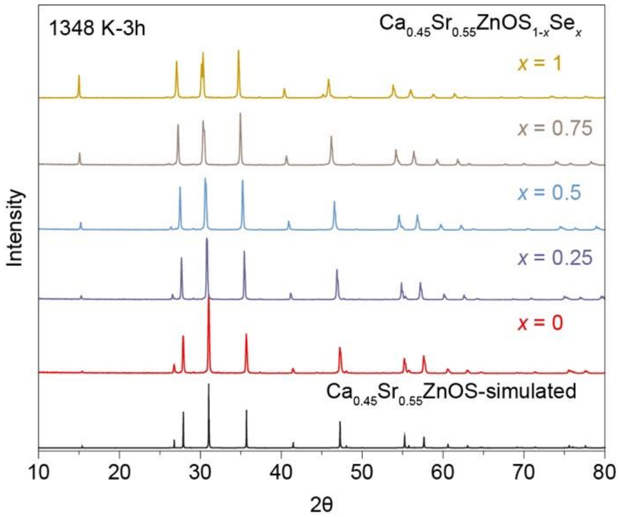

Supplementary Fig. 17. Powder XRD patterns of $Ca_{0.45}Sr_{0.55}ZnOS_{1-x}Se_x$:0.1%$Cu^+$/1%$Y^{3+}$ (x = 0, 0.25, 0.5, 0.75, 1) crystals. The results show the pure hexagonal phase of the samples with various Se contents.

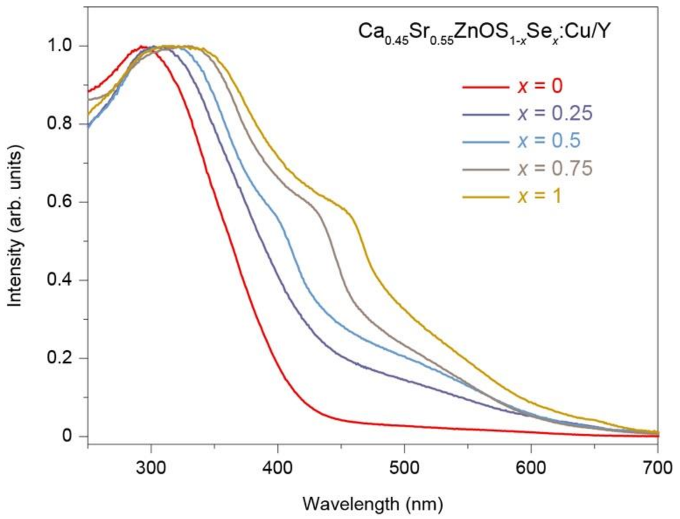

Supplementary Fig. 18. Absorption spectra of the $Ca_{0.45}Sr_{0.55}ZnOS_{1\text{-}x}Se_x$:0.1%$Cu^+$/1%$Y^{3+}$ (x = 0, 0.25, 0.5, 0.75, 1) crystals.

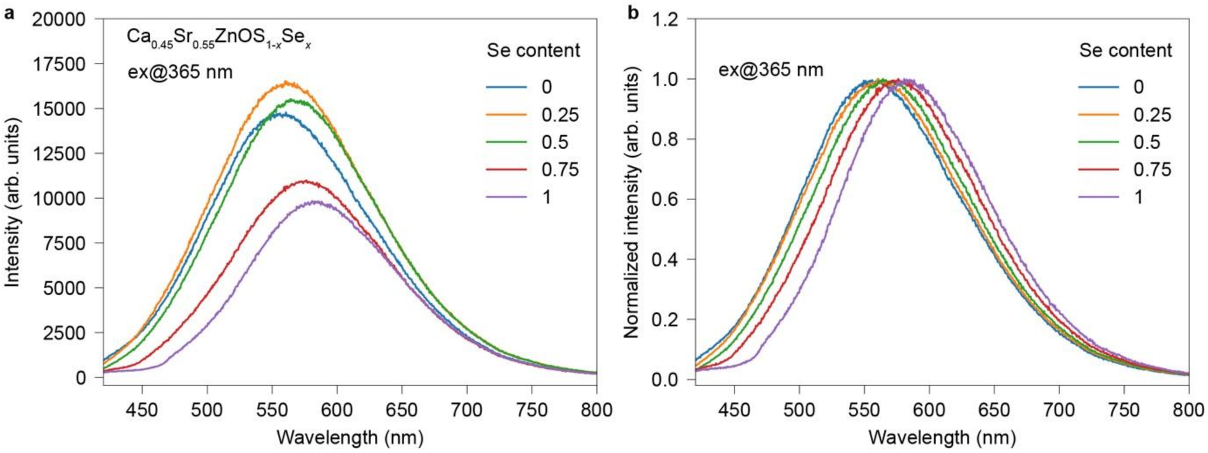

Supplementary Fig. 19. a) PL spectra of the $Ca_{0.45}Sr_{0.55}ZnOS_{1-x}Se_x$:0.1%$Cu^+$/1%$Y^{3+}$ (x = 0, 0.25, 0.5, 0.75, 1) crystals and b) the corresponding normalized PL spectra.

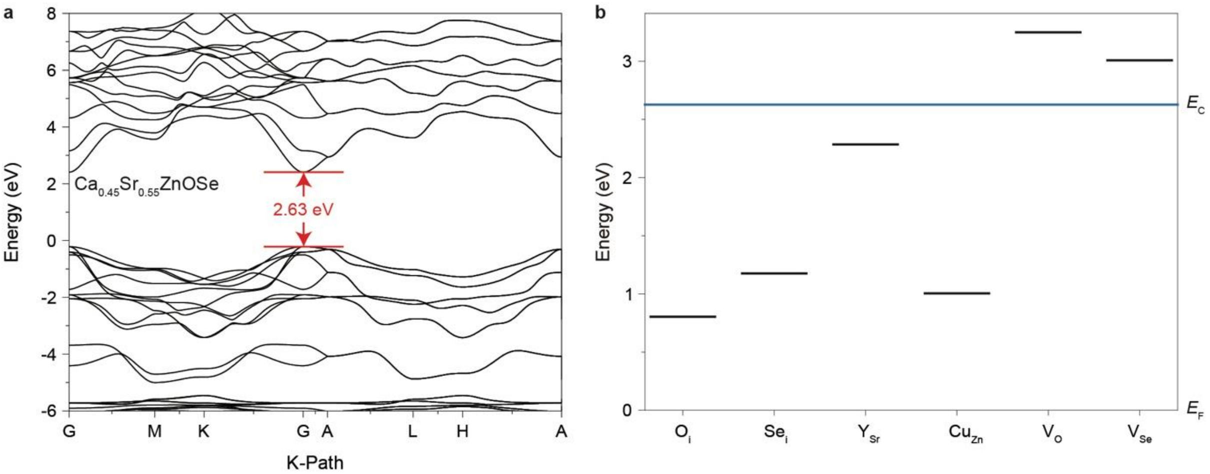

Supplementary Fig. 20. a) Calculated electronic band structures of $Ca_{0.45}Sr_{0.55}ZnOSe$. b) The calculated location of defect states relative to the Fermi level in $Ca_{0.45}Sr_{0.55}ZnOSe$.

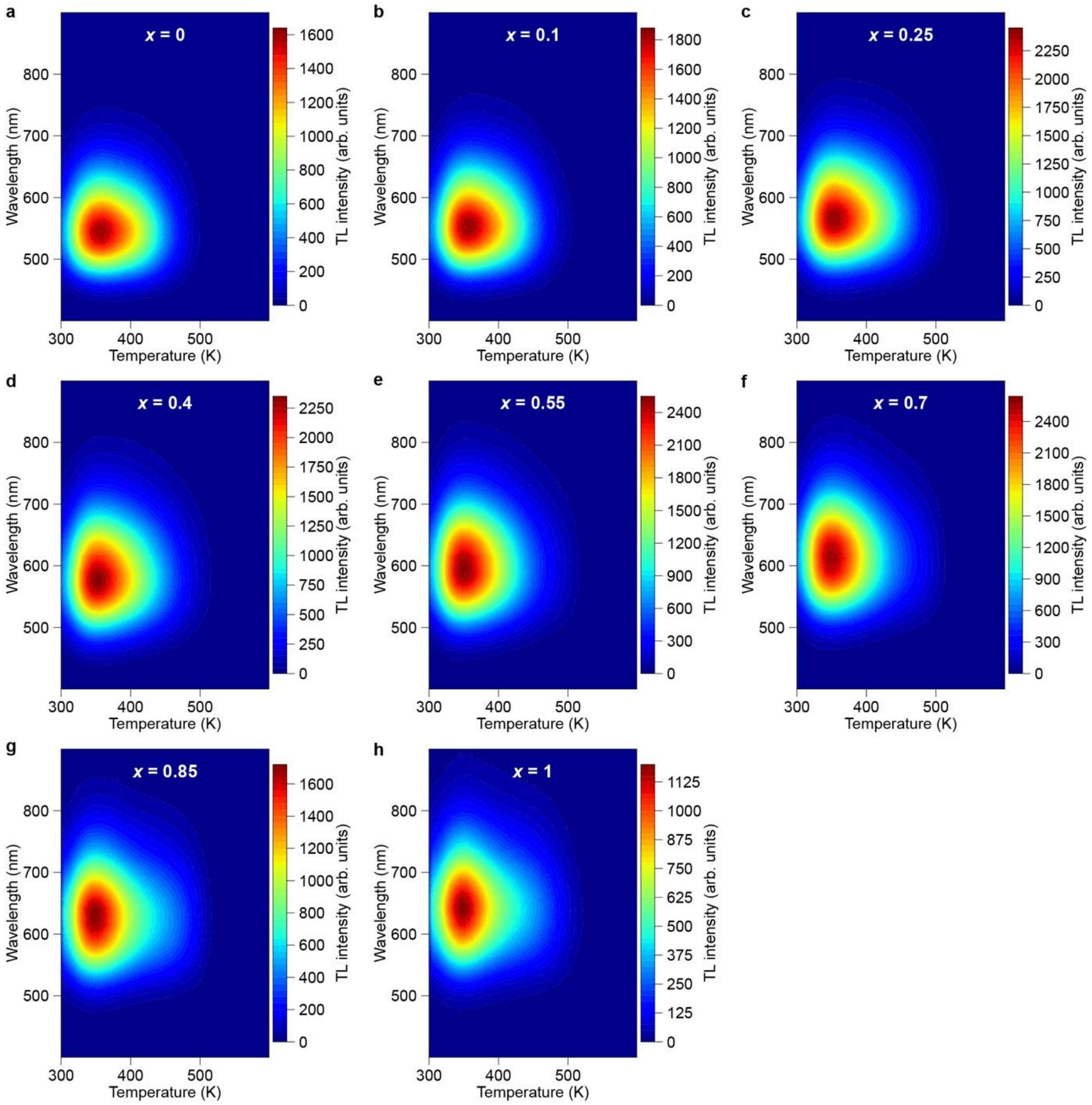

Supplementary Fig. 21. Contour mapping of TL intensity as a function of emission wavelength and temperature for $Ca_{1-x}Sr_xZnOS$:0.1%Cu^+/1%Y^{3+} crystals with various Sr alloying contents: a) $x = 0$, b) $x = 0.1$, c) $x = 0.25$, d) $x = 0.4$, e) $x = 0.55$, f) $x = 0.7$, g) $x = 0.85$ and h) $x = 1$. The heating rate during TL measurement is 1 K s^{-1}. The shift in the emission peak with increasing Sr content aligns with the behaviour observed in room temperature PersL, indicating a similar luminescence nature between TL and PersL.

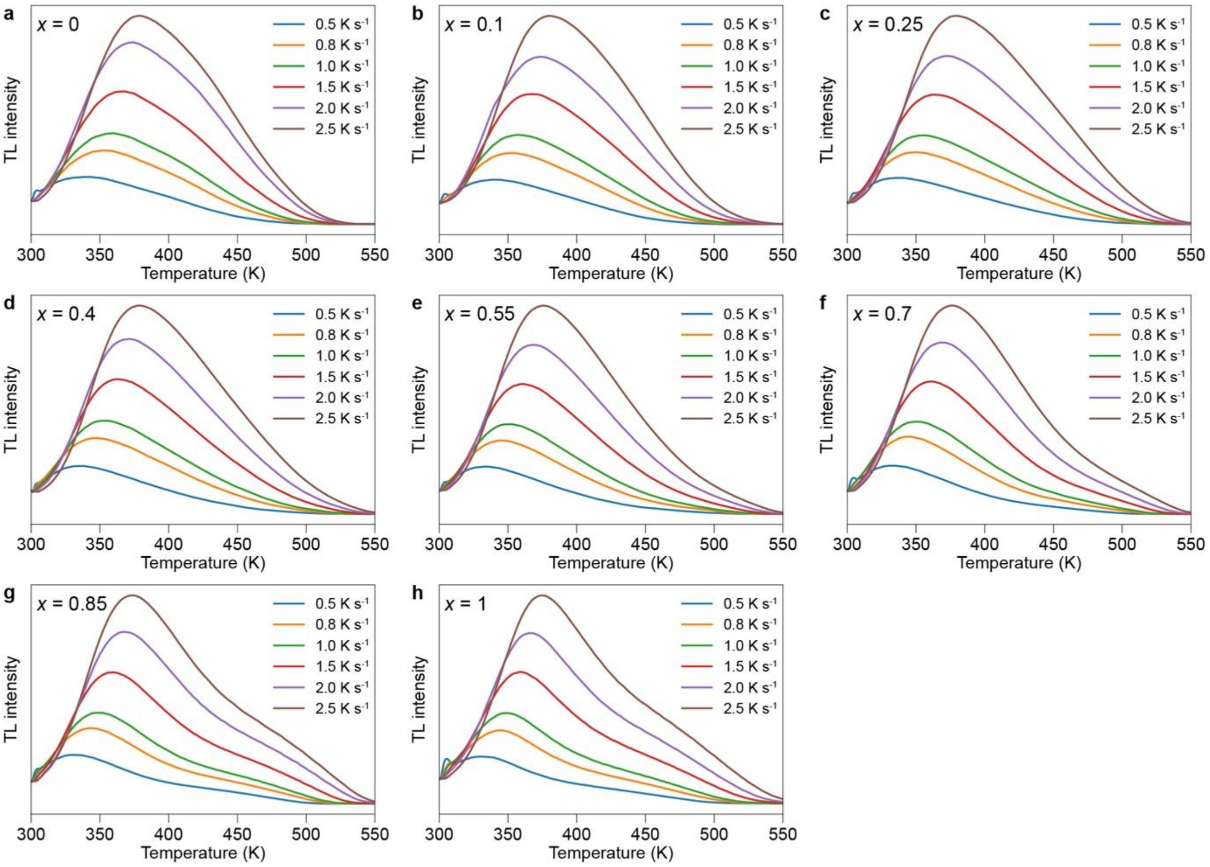

Supplementary Fig. 22. TL spectra recorded at different heating rates for $Ca_{1-x}Sr_xZnOS$:0.1%Cu^+/1%Y^{3+} crystals with various Sr alloying contents: a) $x = 0$, b) $x = 0.1$, c) $x = 0.25$, d) $x = 0.4$, e) $x = 0.55$, f) $x = 0.7$, g) $x = 0.85$ and h) $x = 1$. The samples were pre-charged using a 365 nm UV source (4 W) for 180 s, and a constant delay of 60 s was allowed before each measurement. The heating rate was varied from 0.5 to 2.5 K s^{-1}.

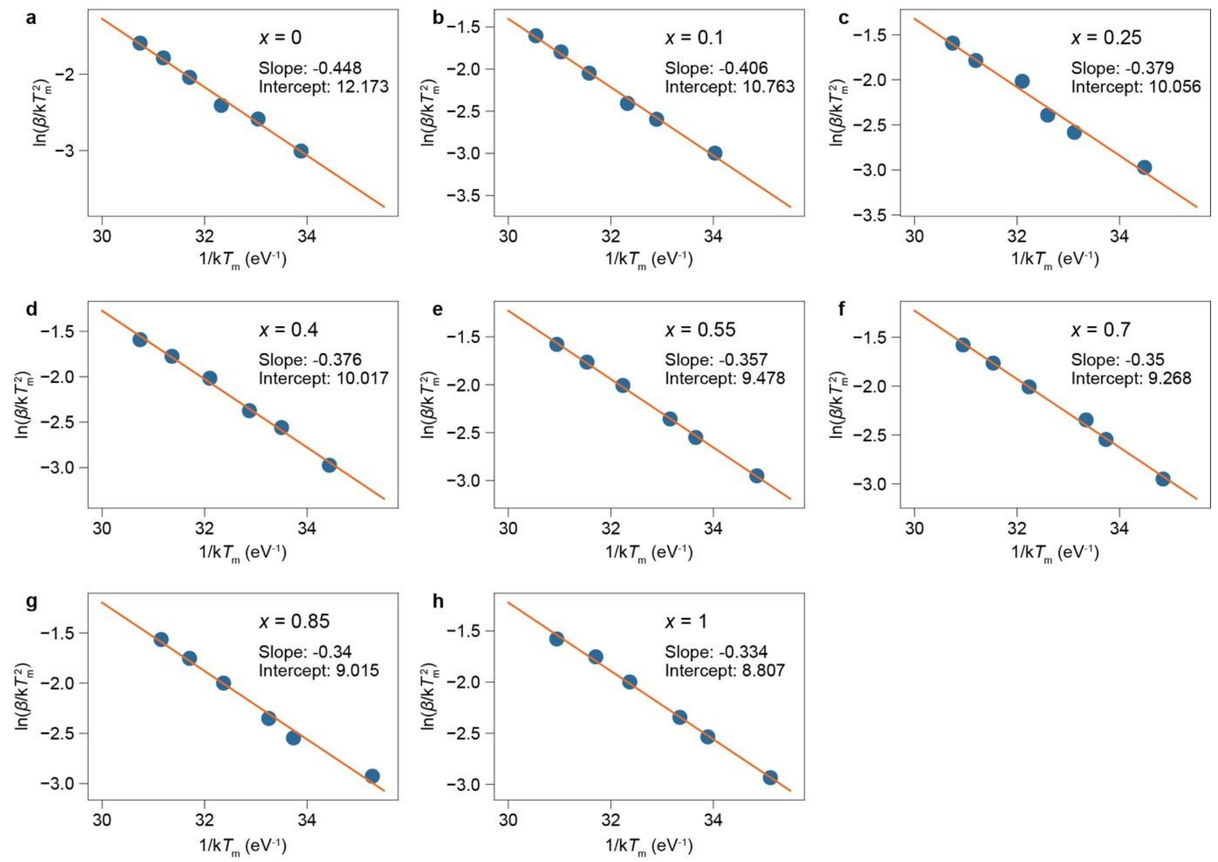

Supplementary Fig. 23. $\ln\left(\frac{\beta}{kT_m^2}\right)$ versus $\frac{1}{kT_m}$ plots as derived from the TL curves for $Ca_{1-x}Sr_xZnOS$:0.1%Cu^+/1%Y^{3+} crystals with various Sr alloying contents: a) $x = 0$, b) $x = 0.1$, c) $x = 0.25$, d) $x = 0.4$, e) $x = 0.55$, f) $x = 0.7$, g) $x = 0.85$ and h) $x = 1$. The heating rate β was varied from 0.5 to 2.5 K s^{-1} and TL peak maximum T_m was determined from the TL glow curves. k is Boltzmann constant. The linear fitting result is included in each subplot.

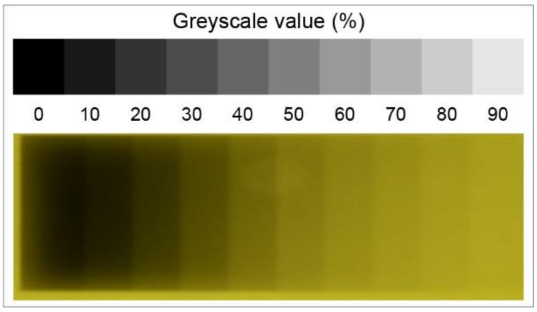

Supplementary Fig. 24. The illustration of the grayscale photomask (top) and the corresponding photograph of the PersL film (bottom) charged in the presence of the photomask.

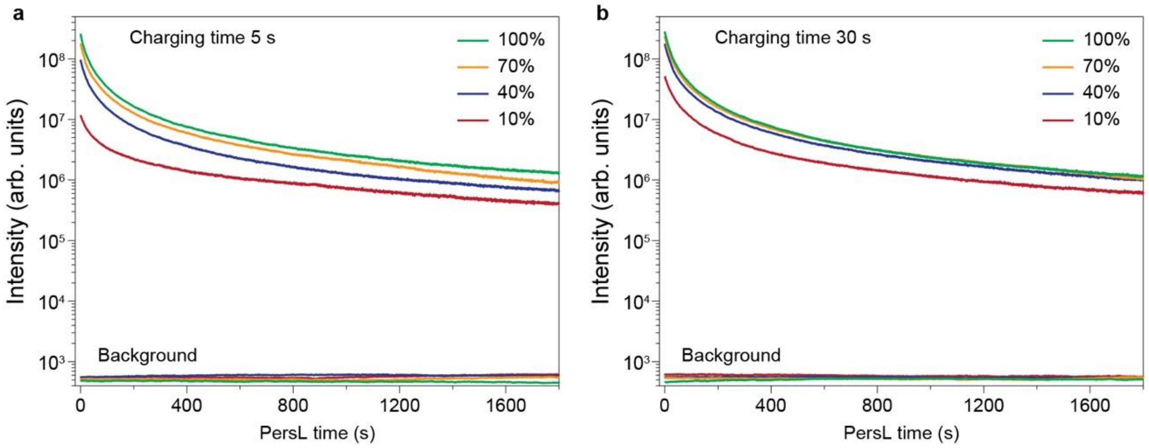

Supplementary Fig. 25. Comparison of PersL decay of the $Ca_{0.45}Sr_{0.55}ZnOS$:Cu/Y sample in the presence of various grayscale masks during charging (100% and 0% stand for total transmission and depletion of charging light), with a charging time of a) 5s and b) 30s, respectively.

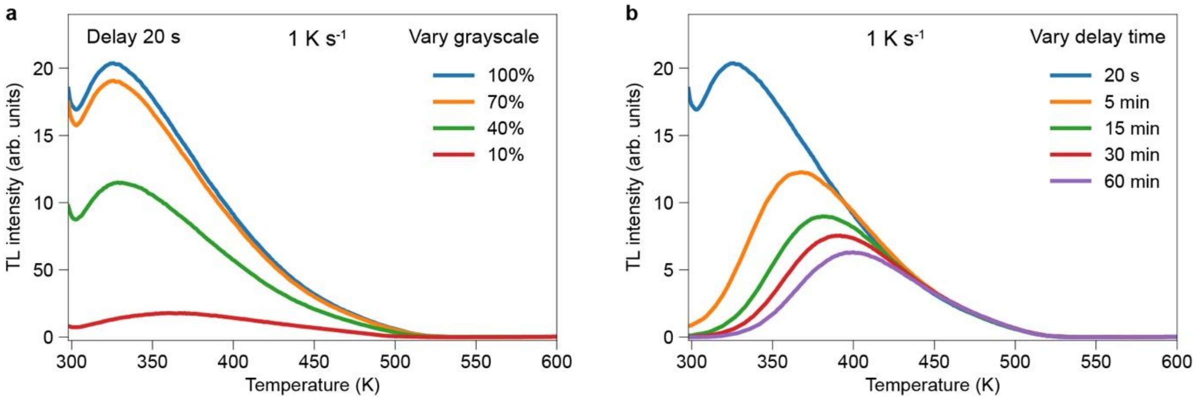

Supplementary Fig. 26. a) TL spectra of $Ca_{0.45}Sr_{0.55}ZnOS$:0.1%Cu^{+}/1%Y^{3+} in the presence of various grayscale masks during charging. The samples were pre-charged using a 365 nm UV lamp (4 W) for 5 s, and a short delay of 20 s was allowed before each measurement. b) TL spectra of $Ca_{0.45}Sr_{0.55}ZnOS$:0.1%Cu^{+}/1%Y^{3+} (charged for 5 s through 100% grayscale photomask) with various delay times before each measurement.

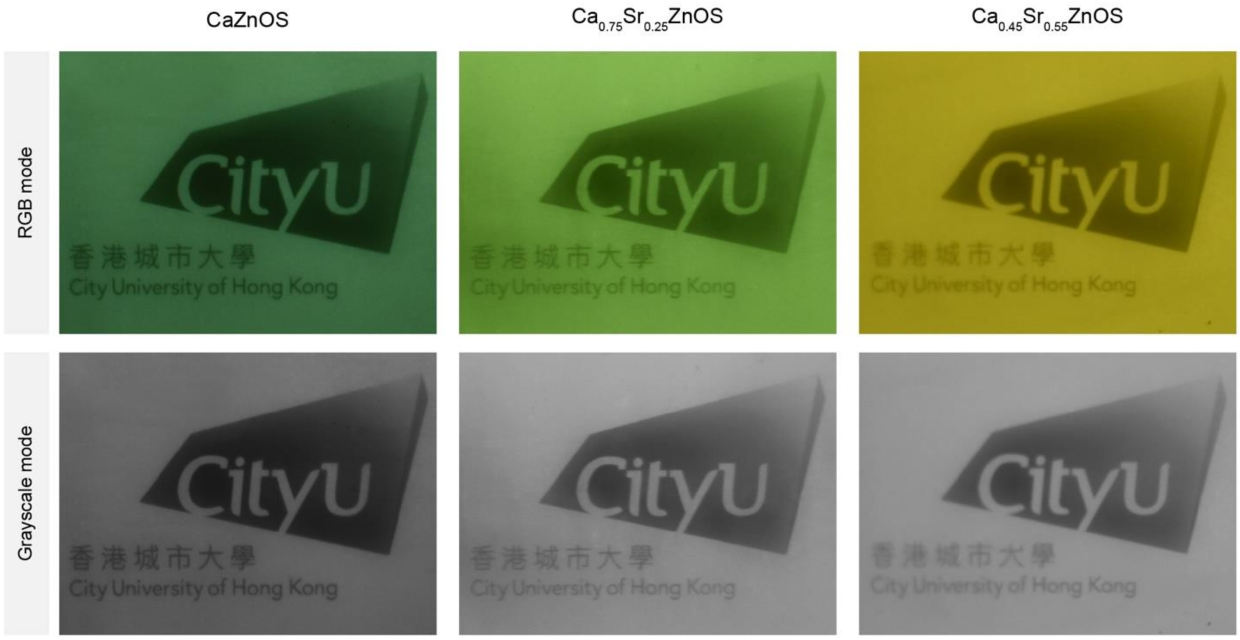

Supplementary Fig. 27. Grayscale patterning based on various Ca(Sr)ZnOS:Cu/Y crystals.

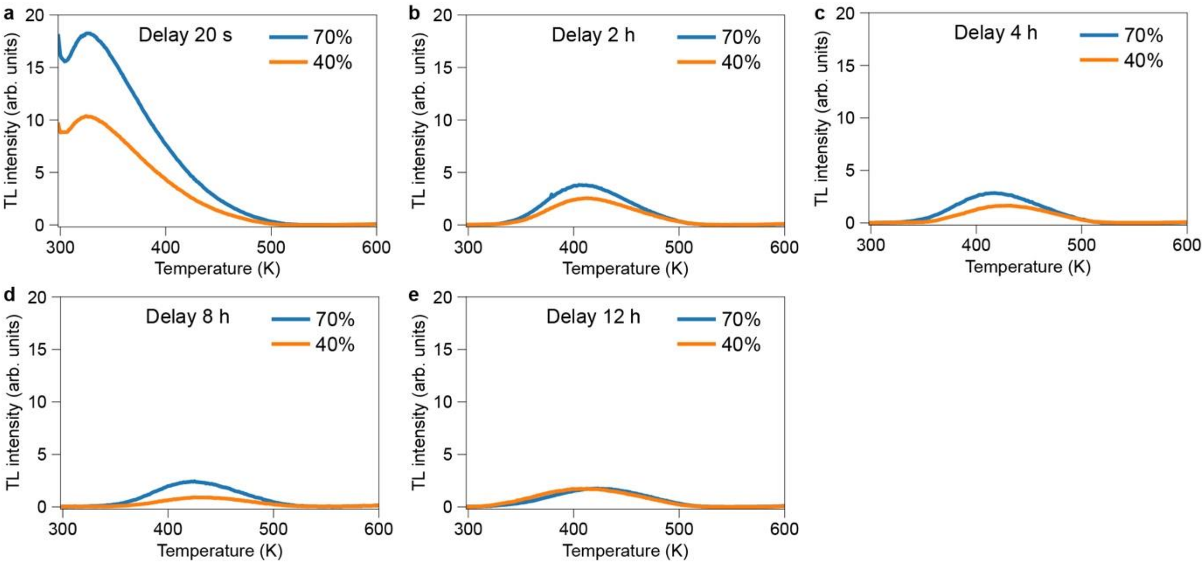

Supplementary Fig. 28. TL spectra of $Ca_{0.45}Sr_{0.55}ZnOS$:0.1%Cu^{+}/1%Y^{3+} (charged for 5 s through 70% or 40% grayscale photomask) with various delay times before each measurement: a) 20 s, b) 2 h, c) 4 h, d) 8 h and e) 12 h.

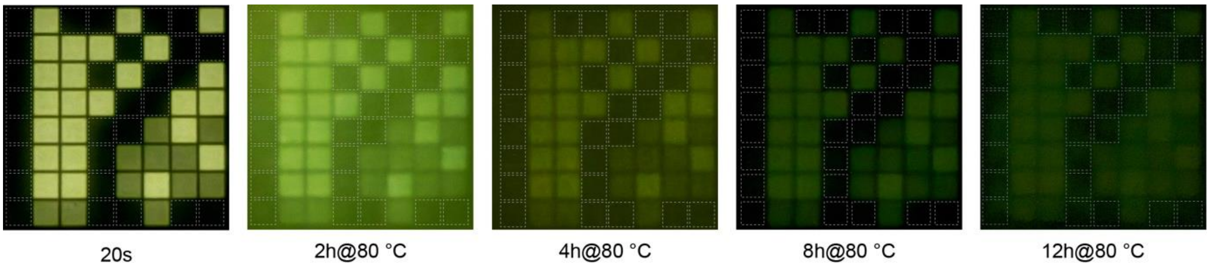

Supplementary Fig. 29. Time evolution of optical information through thermoluminescence readout. The luminescence intensity contrast of partially blocked regions (40/70%) remained distinguishable after an 8-hour delay following charging.

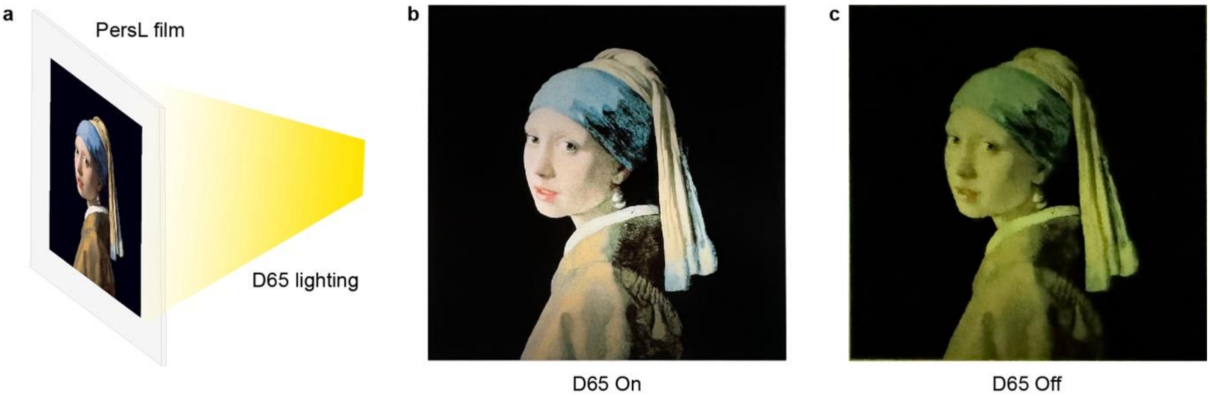

Supplementary Fig. 30. D65 charged multicolor display based on the PersL film containing blended Ca(Sr)ZnOS:Cu^{+}/Y^{3+} crystals. a) Schematic of the illuminating setup. The PersL film was covered by the patterned film on one side, while the D65 light source was positioned on the opposite side for charging. b) Photograph of the display when D65 lamp was turned on. c) Photograph of the display after D65 lamp was turned off.

II. Supplementary Tables

Supplementary Table 1. Brief summary of literature work on PersL spectrum tuning.

Materials	Emissions	Spectrum tunability	Ref.
Polycyclic aromatic hydrocarbons (PAHs)-melamine formaldehyde (MF) composites	466 nm (TP-MF)<br>503 nm (PA-MF)<br>544 nm (FA-MF)<br>592 nm (Py-MF)<br>601 nm (BA-MF)	Discrete	*Angew. Chem. Int. Ed.* **2024**, 136, e202318516
Fluorescein sodium (FluNa)-$Al_2(SO_4)_3$	483, 549 nm	Discrete	*Angew. Chem. Int. Ed.* **2023**, 135, e202217616
Calcein sodium (CalNa)-$Al_2(SO_4)_3$	466, 548 nm	Discrete	*Angew. Chem. Int. Ed.* **2023**, 135, e202217616
MCATMA/C545T	408, 526, 560, 580 and 600 nm	Discrete	*Adv. Mater.* **2022**, 34, 2206712
Cytosine-Cd/ZnX_2 (X = Cl, Br)	435, 513 nm (Cy-$CdCl_2$)<br>435, 513 nm (Cy-$ZnCl_2$)	Discrete	*Adv. Sci.* **2022**, 9, 2200992
Poly(acrylamide-co-N-vinylcarbazole) based host-guest composites	414 nm (Host)<br>502 nm (PBD, guest)<br>560 nm (Fluc, guest)<br>570 nm (Rh123, guest)<br>620 nm (RhB, guest)	Discrete	*Sci. Adv.* **2022**, 8, eabk2925
(R,R)-DAACH/(S, S)-DAACH	470–530 nm	Discrete (Excitation dependent)	*Nat. Commun.* **2022**, 13, 429
4,4′-bis(N-carbazolyl)-1,1′-biphenyl (pCBP)	430, 560 nm	Discrete	*Angew. Chem. Int. Ed.* **2020**, 59, 10032
2,4,6-trimethoxy-1,3,5-triazine (TMOT)	465–505 nm	Discrete (Excitation dependent)	*Nat. Photonics* **2019**, 13, 406
Organic molecules (H-aggregates)	530, 575 nm (DPhCzT)<br>515, 547 nm (DEOPh)<br>529, 574 nm (DECzT)<br>543, 591 nm (CzDCIT)<br>587, 644 nm (DCzPhP	Discrete	*Nat. Mater.* **2015**, 14, 685
Multicomponent copolymer	445, 517 nm (PDNA)<br>445, 514 nm (PDBA)	Discrete	*Nat. Commun.* **2020**, 11, 944
$NaLuF_4$:Ln^{3+}/Gd^{3+} (Ln^{3+}: trivalent lanthanide ion)	489, 546 and 584 nm (Tb^{3+})<br>385 nm (Nd^{3+})<br>453 nm (Tm^{3+})<br>542 nm (Ho^{3+})<br>543 nm (Er^{3+})<br>573 nm (Dy^{3+})<br>594 nm ($Sm^{3+)}$<br>606 nm (Pr^{3+})	Discrete	*Nature* **2021**, 590, 410
Pr^{3+} doped in multiple hosts	UVC ($Sr_2Al_2SiO_7$)<br>266 nm ($Sr_3Y_2Si_6O_{18}$)<br>268 nm ($Ca_2Al_2SiO_7$)<br>267 nm ($Ca_3Al_2Si_3O_{12}$)<br>270 nm (Lu_2SiO_5)<br>267 nm ($LiYSiO_4$)	Discrete	*Nat. Commun.* **2020**, 11, 2040
$LiGa_5O_8$:Mn^{2+} transparent glass ceramic	510, 625 nm	Discrete	*Light Sci. Appl.* **2020**, 9, 22
$CdSiO_3$@SiO_2 nanoparticles	438 nm (In^{3+})<br>438, 580 nm (In^{3+} and Mn^{2+})<br>549 nm (Tb^{3+})	Discrete	*Adv. Mater.* **2020**, 32, 2003881

	578 nm (Dy^{3+})		
$SrSi_2O_2N_2:Ln^{2+}/Ln^{3+}$ (Ln: lanthanides)	620 nm (Yb^{2+}/Dy^{3+},Yb^{2+}/Ho^{3+}, Yb^{2+}/Er^{3+})<br>540 nm (Eu^{2+}/Dy^{3+},Eu^{2+}/Ho^{3+}, Eu^{2+}/Er^{3+})	Discrete	*ACS Appl. Mater. Interfaces* **2018**, 10, 1854
$NaYF_4:Ln^{3+}$@ $NaYF_4$ (Ln: lanthanides)	1525 nm (Er)<br>1475 nm (Tm)<br>1180 nm (Ho)<br>1064 nm (Nd)	Discrete	*Nat. Nanotechnol.* **2021**, 16, 1011
Cs_2CdCl_4 and $Cs_2CdCl_4:Mn^{2+}$	500, 612 nm (Cs_2CdCl_4)<br>600 nm ($Cs_2CdCl_4:Mn^{2+}$)	Discrete	*Angew. Chem. Int. Ed.* **2023**, 62, e202308420
$CaZnOS:Pb^{2+}/Ln^{3+}$ and $CaZnOS:Cu^{+}/Ln^{3+}$	Multiple emission centers	Discrete	*Laser Photonics Rev.* **2023**, 17, 2300132
$CaAl_2O_4:Eu^{2+}Nd^{3+}/CsPbX_3$/PDMS composite (X: Cl^-, Br^-, I^-)	440–694 nm (quantum dots as color converters)	Continuous (complex composite)	*Angew. Chem. Int. Ed.* **2019**, 131, 7017

Supplementary Table 2. The calculated crystallographic structural parameters of the $Ca_{1-x}Sr_xZnOS$:0.1%Cu^+/1%Y^{3+} crystals (x = 0, 0.1, 0.25, 0.4, 0.55, 0.7, 0.85, 1) by Rietveld refinement of the corresponding XRD patterns.

Compound	Space group	*Z*	*a* (Å)	*c* (Å)	Unit cell volume (Å^3)	R_{wp}
CaZnOS	$P6_3mc$	2	3.7574	11.3923	139.287	11.479%
$Ca_{0.9}Sr_{0.1}ZnOS$	$P6_3mc$	2	3.7729	11.4096	140.653	11.823%
$Ca_{0.75}Sr_{0.25}ZnOS$	$P6_3mc$	2	3.7965	11.4412	142.816	11.923%
$Ca_{0.6}Sr_{0.4}ZnOS$	$P6_3mc$	2	3.8208	11.4730	145.047	11.048%
$Ca_{0.45}Sr_{0.55}ZnOS$	$P6_3mc$	2	3.8440	11.5047	147.220	11.161%
$Ca_{0.3}Sr_{0.7}ZnOS$	$P6_3mc$	2	3.8675	11.5384	149.461	11.129%
$Ca_{0.15}Sr_{0.85}ZnOS$	$P6_3mc$	2	3.8878	11.5693	151.444	10.795%
SrZnOS	$P6_3mc$	2	3.9096	11.6059	153.632	11.777%

Supplementary Table 3. The brightness of the newly developed Ca(Sr)ZnOS PersL materials (charged by a 4 W 365 nm handheld UV lamp).

	Brightness (cd m^{-2})			
	5 s	20 s	30 s	1 min
CaZnOS:Cu/Y	3.49	0.75	0.45	0.20
$Ca_{0.9}Sr_{0.1}ZnOS$:Cu/Y	3.63	0.96	0.63	0.29
$Ca_{0.75}Sr_{0.25}ZnOS$:Cu/Y	4.48	1.00	0.64	0.30
$Ca_{0.6}Sr_{0.4}ZnOS$:Cu/Y	5.36	1.14	0.72	0.33
$Ca_{0.45}Sr_{0.55}ZnOS$:Cu/Y	5.07	0.98	0.64	0.28
$Ca_{0.3}Sr_{0.7}ZnOS$:Cu/Y	4.03	0.86	0.54	0.25
$Ca_{0.15}Sr_{0.85}ZnOS$:Cu/Y	1.90	0.42	0.29	0.13
SrZnOS:Cu/Y	1.57	0.30	0.19	0.08

Supplementary Table 4. The brightness of the newly developed Ca(Sr)ZnOS PersL materials (charged by an 18 W D65 lamp).

	Brightness (cd m^{-2})				
	5 s	10 s	20 s	30 s	1 min
CaZnOS:Cu/Y	0.48	0.28	0.17	0.13	0.09
$Ca_{0.9}Sr_{0.1}ZnOS$:Cu/Y	0.66	0.40	0.23	0.18	0.10
$Ca_{0.75}Sr_{0.25}ZnOS$:Cu/Y	1.27	0.74	0.41	0.30	0.17
$Ca_{0.6}Sr_{0.4}ZnOS$:Cu/Y	1.97	1.04	0.55	0.39	0.21
$Ca_{0.45}Sr_{0.55}ZnOS$:Cu/Y	2.11	1.12	0.62	0.45	0.25
$Ca_{0.3}Sr_{0.7}ZnOS$:Cu/Y	1.59	0.85	0.46	0.34	0.19
$Ca_{0.15}Sr_{0.85}ZnOS$:Cu/Y	0.86	0.44	0.24	0.17	0.11
SrZnOS:Cu/Y	1.39	0.63	0.31	0.21	0.11

Supplementary Table 5. Comparison of the PersL peak (λ_{max}) of various activators in CaZnOS and SrZnOS.

Activator	λ_{max} in CaZnOS (nm)	λ_{max} in SrZnOS (nm)	λ_{max} shift (nm)	Remarks
Mn^{2+}	613	615	2	Minor crystal field change
Tb^{3+}	544	544	0	Dual emissions of D-A and Tb^{3+}
Yb^{3+}	982	983	1	4f-4f transition
Bi^{3+}	466	483	17	$6s^2$-6s6p transition
Pb^{2+}	425	500	75	Self-trapped excitons
Cu^{+}	501	549	48	
**Cu^{+}-Y^{3+}**	**527**	**630**	**103**	

Supplementary References

1 Zhang, X. *et al.* Systematic Tuning of Persistent Luminescence in a Quaternary Wurtzite Crystal Through Synergistic Defect Engineering. *Laser Photonics Rev.* **17**, 2300132 (2023).
